# Spatial patterning of the Notch ligand Dll4 controls endothelial sprouting *in vitro*

**DOI:** 10.1038/s41598-018-24646-y

**Published:** 2018-04-23

**Authors:** L. A. Tiemeijer, J-P. Frimat, O. M. J. A. Stassen, C. V. C. Bouten, C. M. Sahlgren

**Affiliations:** 1 Department of Biomedical Engineering, Soft Tissue Engineering and Mechanobiology, ICMS Institute for Complex Molecular Systems, Eindhoven University of Technology, Eindhoven, Netherlands; 2 Faculty for Science and Engineering, Biosciences, Åbo Akademi University, Turku, Finland; 3 Department of Mechanical Engineering, Microsystems Group, Eindhoven University of Technology, Eindhoven, Netherlands; 4 Turku Centre for Biotechnology, University of Turku and Åbo Akademi University, Turku, Finland

## Abstract

Angiogenesis, the formation of new blood vessels, is a vital process for tissue growth and development. The Notch cell-cell signalling pathway plays an important role in endothelial cell specification during angiogenesis. Dll4 - Notch1 signalling directs endothelial cells into migrating tip or proliferating stalk cells. We used the directing properties of Dll4 to spatially control endothelial cell fate and the direction of endothelial sprouts. We created linear arrays of immobilized Dll4 using micro contact printing. HUVECs were seeded perpendicular to these Dll4 patterns using removable microfluidic channels. The Notch activating properties of surface immobilized Dll4 were confirmed by qPCR. After induction of sprouting, microscopic images of fluorescently labelled endothelial sprouts were analysed to determine the direction and the efficiency of controlled sprouting (Ecs). Directionality analysis of the sprouts showed the Dll4 pattern changes sprout direction from random to unidirectional. This was confirmed by the increase of Ecs from 54.5 ± 3.1% for the control, to an average of 84.7 ± 1.86% on the Dll4 patterned surfaces. Our data demonstrates a surface-based method to spatially pattern Dll4 to gain control over endothelial sprout location and direction. This suggests that spatial ligand patterning can be used to provide control over (neo) vascularization.

## Introduction

Since diffusion is not efficient over larger distances, blood vessels are crucial for transport of nutrients, oxygen and elimination of waste to support organ growth and repair of damaged tissue^1^. Inefficient transport leads to tissue hypoxia and a demand for the formation of new blood vessels to restore the blood supply and stabilize local oxygen levels. It is known that under the influence of vascular endothelial growth factor (VEGF) released by hypoxic or injured tissue, new vessels sprout from existing vessels, a process called angiogenesis^2,3^. In angiogenic sprouting, one endothelial cell (tip cell) guides the other (stalk) cells towards the VEGF gradient. Delta like ligand 4 (Dll4) – Notch 1 signalling has an important role in sprouting angiogenesis^2–4^. Dll4 – Notch 1 is involved in the specification of endothelial cells (ECs) into migratory tip or proliferating stalk cells^3,5–7^. The binding of VEGF-A to vascular growth factor receptor 2 (VEGFR2) induces Dll4 expression in all endothelial cells^2^. However, some ECs express more extracellular Dll4 than others, thereby inducing Notch signalling in the neighbouring ECs and a stalk cell phenotype, while the Dll4 expressing EC adopts a tip cell phenotype^2^. Induction of Notch signalling in the neighbouring cells reduces expression of VEGFR2 and induces expression of VEGFR1, which desensitizes the cell for the VEGF-A gradient^8,9^. This interplay of the Dll4 - Notch 1 signalling with the VEGF-A gradient has been suggested to be responsible for a salt and pepper pattern of tip- and stalk endothelial phenotypes^10^. The tip and stalk phenotypes are not stable, but reside in a dynamic state and constantly interchange in response to changing Notch signalling levels within individual cells^11^. The influence of Notch signalling on angiogenesis has been studied mainly from a biology driven perspective by confounding techniques. These include *in vivo* knock-in, knock-out models which are influenced by systemic factors, *in vitro* co-cultures influenced by the other cell source, the usage of non-specific Notch signalling inhibitors like DAPT or ligand specific decoys^5–7,9,12,13^.

**Figure 1:**
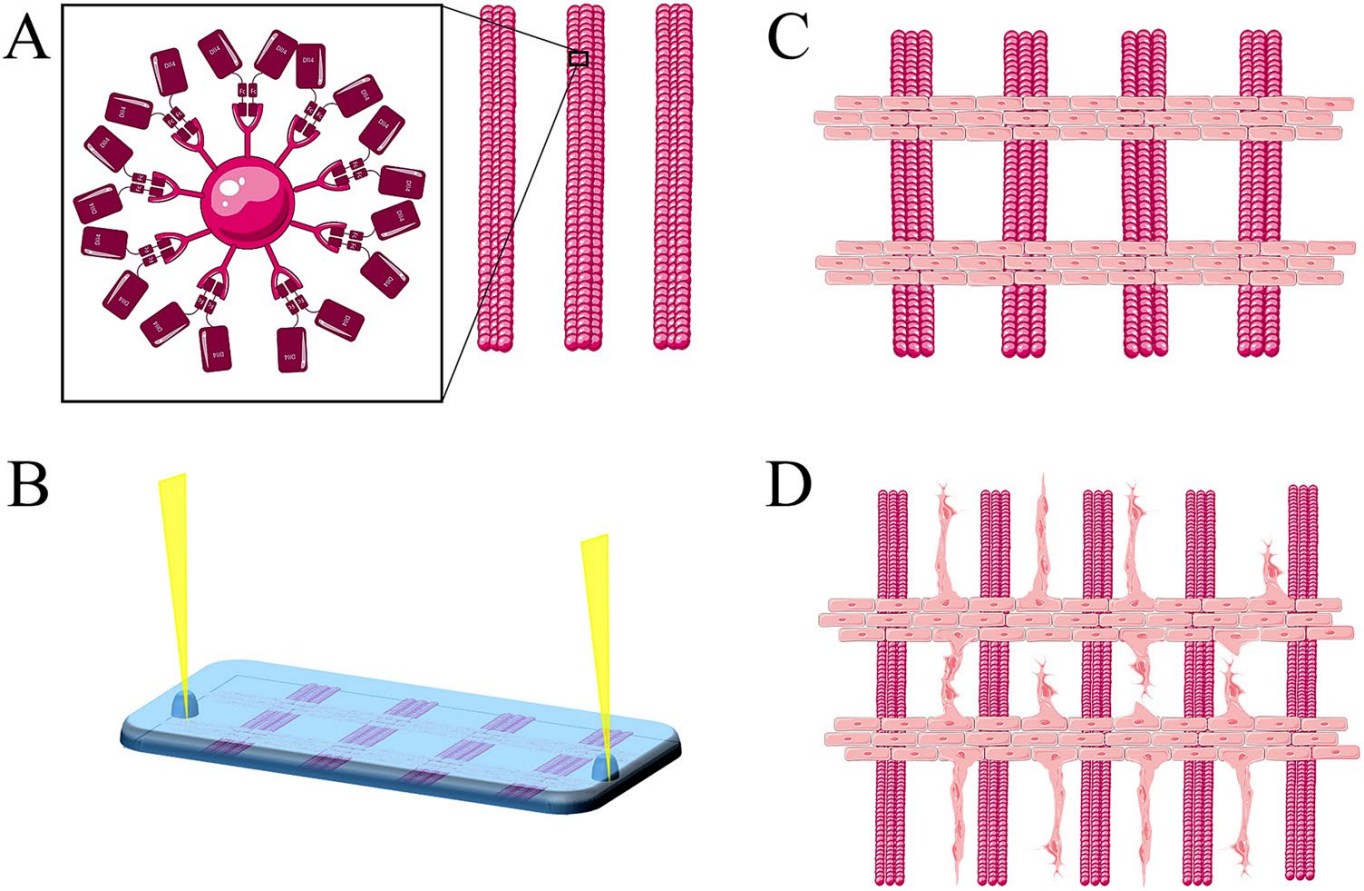
Schematic overview of the method described in this study. In (**A**) patterning of Dll4 by micro contact printing of the Dll4-fc immobilized to pink fluorescent beads in parallel lines with defined width and spacing. In (**B**) seeding of HUVECs through microfluidic channels perpendicular on the patterned Dll4. In (**C**) an overview of the HUVECs in (vessel mimic mimicking) monolayers perpendicular on top of the patterned Dll4 after removal of the microfluidic channels. (**D**) Hypothesized sprouting of the HUVECs after 24 h culture in Matrigel enriched media. Images made with tools from Servier medical art by Servier.

Here we addressed the question if sprouting angiogenesis can be externally controlled by exposure to patterns of Notch ligands. Such external control is of interest in tissue engineering to control and enhance efficient vascularization of tissue-engineered constructs. Notch signalling is inhibited by soluble ligands, but Notch signalling can be activated *in vitro* by immobilization of recombinant Notch ligands^9,14,15^. So far, the induction of Notch signalling *in vitro* using immobilized ligands has been done on homogenously coated (bio)materials^16–19^. Since Notch signalling is cell contact dependent and Dll4 signalling operates through lateral induction e.g. inducing the signal receiving cells to adopt a different fate than the Dll4 expressing signal sending cell, the location of the Dll4 induction should influence the endothelial response^2,4,8^. Therefore, manipulation of Dll4 – Notch 1 signalling to control vascular patterning is of interest. Endothelial cell fate is determined by the local higher concentration of signalling Dll4 originated from the neighbouring cell^2^. To recreate and manipulate this, a surface was created where the high Dll4 signalling neighbour was mimicked by a printed pattern, to see whether we could force the cells on top of this Dll4 pattern to adopt a stalk cell phenotype, leaving the adoption of a tip cell phenotype to the cells in between the high Dll4 sites in the pattern. We expected that this would result in the initiation of endothelial sprouting in between the Dll4 patterned lines, thereby gaining control over the location and direction of sprouting. We used microfabrication techniques (Fig. 1) to produce patterns of immobilized Dll4. We used micro contact printing (µCP) to print a pattern of 100 µm wide lines of immobilized and fluorescent Dll4 on a glass surface. These lines represent a local higher concentration of Dll4 adjacent to a Dll4-free surface area. Thereafter, Human Umbilical Vein Endothelial Cells (HUVECs) were seeded perpendicular to these Dll4 lines, using removable micro channels, which allowed for a control of the initial location of the endothelial cells mimicking a monolayer vessel from which sprouting normally occurs. Following release of the channels the endothelial cells were left to sprout in Matrigel enriched media for 24 h. After fixation, immunofluorescence staining and microscopy, the samples were subdued to image analysis to evaluate if the alternating Dll4 pattern dictated sprouting organization. This proof of concept study is the first to demonstrate the possibility of manipulating angiogenesis by using microfabrication techniques to spatially pattern the Notch ligand Dll4 and control direction of sprouting.

## Material and Methods

### Fabrication of micro contact printing (µCP) stamps and microfluidic channels

**Figure 2:**
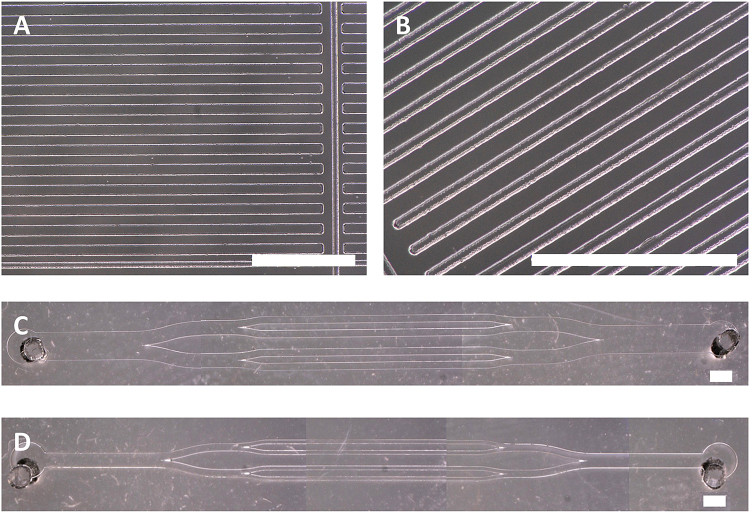
Microscopic images of the different µCP stamps and microfluidic channels. (**A**) Stamps with lines 100 µm wide with 100 µm spacing. (**B**) Stamps with lines 100 µm wide with 50 µm spacing. (**C**) Microfluidic channels with 300 µm wide subchannels. (**D**) Microfluidic channels with 150 µm wide subchannels. Scale bar represents 1 mm.

Standard soft photolithography was used to create SU-8 masters (MicroChem) for both the µCP stamps and the microfluidic channels. Briefly, Su-8 2050 was spun at 500 rpm for 10 s, followed by 30 s at 2000 rpm, prebaked 5 min at 65 °C and 15 min at 95 °C. UV exposure was performed with a mask containing the desired features for 35 s at 13mNcm^−2^. Post baking 5 min at 65 °C and 8 min at 95 °C and finally developed for an hour with SU-8 developer. PDMS consisting of 10:1 w/v parts base to curing agent (Sylgard 184) was degassed by centrifuging and poured onto the SU-8 masters. Secondary degassing under vacuum was followed by curing at 65 °C overnight before PDMS was peeled off to be cut into stamps or channels. µCP stamps were designed to contain straight lines with widths of 100 and 50 µm spaced by 100 µm (Fig. 2A,B). Various microfluidic channels were designed, with channels width of 150 µm and 300 µm, spaced by 300 or 500 µm, with 1.5 mm inlets and outlets (Fig. 2C,D).

### Cell culture

Human Umbilical Vein Endothelial Cells (HUVECs, Lonza) were cultured in Endothelial Base Medium (EBM-2, Lonza) with additives of 2% FBS, 0.04% Hydrocortisone, 0.4% hFGF-B, 0.1% VEGF, 0.1% R3-IGF-1, 0.1% ascorbic acid, 0.1% hEGF, 0.1% GA-1000, and 0.1% heparin (Lonza) supplemented with 1% penicillin/streptomycin in cell culture flasks coated with 0.1% gelatin (porcine, Sigma) at 37 °C, 5% CO_2_. Passage numbers 3–6 were used in these experiments.

### Patterning of Dll4 ligands by µCP

50 µl (10 µg/ml) of active human Dll4 protein fragment ab108557 (Abcam) was incubated with 100 µl (0.1% w/v) protein G fluorescent particles (Spherotech, purple, 0.4–0.6 µm) for 1 h at RT, resulting in a maximum of 0.5 µg of Dll4 ligand immobilized to the beads per sample. Before inking the µCP stamps with 150 µl of the Bead-Dll4 mixture, the stamps were made hydrophilic by treatment with atmospheric plasma for 9 s (Corona Discharge Generator, Tantec HF). In control experiments the stamps were inked with 100 µl of fluorescent beads, without the Dll4 ligands. µCP stamps and ink were incubated for 30 min and dried using N_2_ gas prior to printing on glass slides, which were sterilized with 70% ethanol prior to printing. The µCP stamps were left to adhere to the glass for 30 min at RT before removal.

### Microfluidic patterning

Microfluidic channels were deposited perpendicular to the patterned glass slides by hand (microchannels face down) and gently pressed to form a contact with the substrate to seal the microchannels. P200 pipette tips (without filter) were placed in the inlets of the channels to act as fluidic reservoirs that allowed feeding of the system by gravity (Fig. 1B). The channels were coated with 1% gelatin in PBS, pipetted in one of the tips, for 5 min at 37 °C, 5% CO_2_, followed by careful flushing out of the gelatin solution with preconditioned media (18 h at 37 °C, 5% CO_2_). HUVECs in preconditioned media (9*10^6^ cells/ml) were seeded directly in the pipette tips. The HUVECs were left to adhere for 3 h at 37 °C, 5% CO_2_ while being monitored for cell aggregates and sufficient media flow through the channels. After adherence the channels were gently flushed to wash unattached HUVECS away and media was deposited around the channels before the microchannels were taken off, and was then enriched with Matrigel (15% in total amount of media, Corning, lotnr. 4321005) on top of the HUVECs. The HUVECs were left to sprout for 24 h in 37 °C, 5% CO_2_.

### Fluorescent microscopy and image analysis

The cell samples were fixated for 30 min at RT with 3.7% formaldehyde in PBS. The fixed samples were permeabilised with 0.5% triton X-100 in PBS for 15 min. Netgel (50 mM Tris pH 7.5, 150 mM NaCl, 1 mM EDTA, 0.1% NP-40 (Non-idet) and 0.25% gelatin, in milliQ water) was used as washing buffer. After blocking (30 min, 4% horse serum), the samples were incubated with Dll4 goat anti-human (Santa Cruz) primary antibody at 4 °C overnight. Incubation with IgG donkey anti-goat Alexa555 (Invitrogen) secondary antibody was done for 1 h at RT. Phalloidin (Atto488) and DAPI were used as markers for the actin cytoskeleton and the nuclei respectively. Image acquisition was done using a confocal, two-photon laser scanning Leica TCS SP5X microscope.

The efficiency of patterning the sprouts in the hypothesized area in between the Dll4 lines was quantified by using equation 1 (adjusted from Frimat *et al*.^20^);

1 $$Ecs=\frac{Coff}{(Coff+Con)}100 \% $$

where Ecs is the efficiency of the control over the sprouting of the endothelial cells in percentage, C_off_ is the number of cells that has sprouted from the HUVEC monolayer to the area in between the lines of bead-Dll4 and the C_on_ is the number of cells that have sprouted to the area where they are exposed to the bead-Dll4 complexes. One region of interest (ROI) contains both areas. The direction analysis was done with the image J plugin ‘Directionality’ on multiple ROI’s at once.

### qPCR

Activation of the Notch pathway by the µCP ink was verified with qPCR. Culture plates with 2.5*10^4^ cells/cm^2^ were subjected to different conditions; mimicking the experimental setup µCP ink coated with gelatin and topped with HUVECs in Matrigel enriched media, a control condition where Dll4 was not attached to the beads, and a control where no µCP ink was present. Additionally all conditions were subdued to the Notch signalling inhibitor DAPT (2.5 mg/ml in media, DMSO as vehicle), and its vehicle DMSO as well. After 24 h at 37 °C, 5% CO_2_ RNA was isolated using RNeasy mini kit (Qiagen) according to manufacturer’s instructions. RNA was stored at −30 °C before cDNA synthesis. 200ng RNA per sample was used to synthesize cDNA. cDNA (1ng/µl, 2.5 µl volumes) was amplified using primers for FLT1 (VEGFR1) (Forward (Fw): CAAATAAGCACACCACGCCC, Reverse (Rv); CGCCTTACGGAAGCTCTCTT) KDR (VEGFR2) (Fw: CGGTCAACAAAGTCGGGAGA, Rv: CAGTGCACCACAAAGACACG), FLT4 (VEGFR3) (Fw: TGAGAGACGGCACAAGGATG, Rv: CTCCACCAGCTCCGAGAATG), EFNB2 (Fw: CTGCTGGGGTGTTTTGATGG, Rv: GTACCAGTCCTTGTCCAGGTAG), and references genes B2M and ACTB (Primerdesign) using the iQ Thermal cycler (BioRad). iQ SYBR Green (BioRad) was used as fluorescent dye. The number of experimental repetitions was 3 and the PCR was performed 3 times in total.

### Statistical analysis

Data was presented as mean ± standard error of the mean (SEM). A one way non-parametric ANOVA Kruskal-Wallis with a Dunn’s multiple comparison post hoc test was done to analyse the directionality data, and some of the Ecs data. A non-parametric student-t Mann-Whitney test was used to compare one data set of the Ecs data. A parametric ANOVA with a Bonferoni multiple comparison post hoc test for selected columns was used for the qPCR data. All statistics were done using the GraphPad Prism software. A difference with a p-value less than 0.05 was considered significant.

• The datasets generated during and/or analysed during the current study are available from the corresponding author upon request.

## Results and Discussion

### Immobilized Dll4 activates endothelial Notch signalling

**Figure 3:**
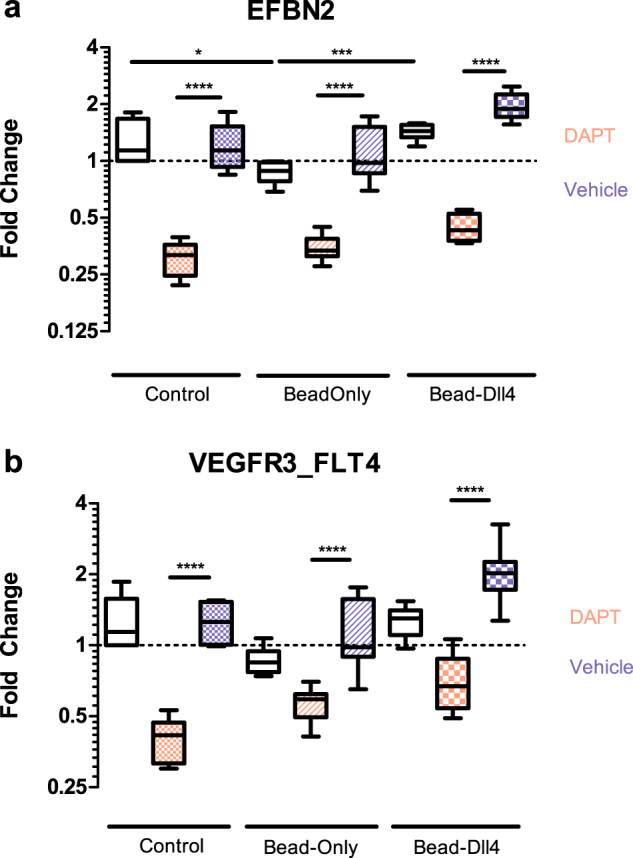
Fold change in gene expression of Ephrin B2 en Flt4 (VEGFR3). The experimental conditions are indicated below the graphs and in the legend adjacent. The condition of Beads with or without attached Dll4 underneath a gelatin coating is used here as well as the rest of this study. DMSO was used as a vehicle control for DAPT. Experiment in triple, qPCR was repeated 3 times. *p < 0.05, **p < 0.01, ***p < 0.001, ****p < 0.0001.

We generated a homogenously Dll4 functionalized surface on regular plastic culture plates to verify that the immobilized bead-Dll4 complexes were able to activate Notch signalling. Expression of endothelial specific Dll4 – Notch 1 induced target genes was analysed by qPCR on samples of all the experimental conditions and controls. In addition to verifying if the bead Dll4 complex can sustain Dll4 – Notch 1 signalling (Fig. 3), the experiment assessed if induction of Notch signalling by Dll4 through a gelatin coat is possible, as well as investigated the impact of the naked IgG beads on Notch target gene expression. The interaction of Dll4 – Notch 1 and activation of Notch signalling in the receiving (stalk) cell leads to a feedback loop where the expression of VEGF-receptors (VEGFR) are either induced or reduced depending on the receptor^8^. Dll4 induced activation of Notch is thought to repress FLT1 (VEGFR1) expression and promote expression of FLT4 (VEGFR3). KDR (VEGFR2) as a Dll4 – Notch 1 signalling target gene is still debated^21^. We also included the Notch target gene Ephrin B2 in the analyses^22^. Dll4 – Notch 1 signalling was pharmacologically inhibited by the Notch inhibitor DAPT.

The expression of Ephrin B2 and FLT4 (VEGFR3) in HUVECs cultured on top of empty beads, bead-Dll4 complexes and control, was significantly decreased upon treatment with DAPT, indicating that active (basal) Dll4 – Notch 1 signalling is present in the endothelial cell layer. Additionally, Ephrin expression was increased upon the addition of Dll4 to the beads, indicating the immobilization of Dll4 to the fluorescent beads enhances Dll4 – Notch 1 signalling of the cells exposed to the bead-Dll4 complexes. A similar trend can be observed in the expression of VEGFR3, however this was not found significant. It must be noted that the basal Notch activity in these cultures are high, as the cells are grown at high confluency and are in contact with each other, resulting in a constant Dll4 - Notch 1 signalling feedback loop which involves the VEGF receptors. This might explain why a trend of increased VEGFR3 expression can be observed as a result of the bead-Dll4 complexes, but cannot be found to make a significant difference for this particular target gene. Importantly, the beads alone did not induce the expression of key angiogenic target genes and immobilized bead - Dll4 was still able to activate Notch 1 signalling after gelatin coating as seen in the data of Ephrin gene expression.

### Patterning recombinant Dll4 ligands by microfabrication techniques

A microfabrication based platform to control endothelial sprouting was designed using µCP to pattern immobilized Dll4 (bead-Dll4) and microfluidic channels to control the seeding and alignment of HUVECs on the patterned Dll4. This approach enabled us to exclude anti-fouling agents to allow free cellular movement after release of the microfluidic channels. This way, endothelial sprouts were constrained and influenced by the experimental conditions alone, and all resulting control over migration, proliferation or sprouting was induced by the micro contact printed bead-Dll4 pattern. During data analyses, regions of interest (ROIs) were defined as regions containing a clearly visible line of patterned fluorescent beads (either linked to Dll4 or not) and the same area in between two lines, alongside a proper endothelial cell monolayer mimicking the endothelial lining in (micro) vessels. Matrigel provided an extracellular matrix (ECM) mimicking structure, which together with additional growth factors in the media, supported endothelial sprouting^23^. µCP as used in this study relies on manual reproducibility. Therefore, a maximum of 0.5 µg of Dll4 ligand immobilized to fluorescent beads per sample could be reached. This amount of ligand corresponds to the range used in previous experiments in our group^24,25^. Notch signalling is inhibited by soluble ligands^9,14^, and immobilization of ligands has been previously been proven to be necessary to induce Notch signalling in cells and tissue^14,15^. Therefore, we believe this *in vitro* surface based Dll4 – Notch 1 inducing patterning method might mimic cell-cell signalling, and could be used to override natural cell-fate deciding signalling.

### Printed bead-Dll4 patterns lead to patterned endothelial sprouts

**Figure 4:**
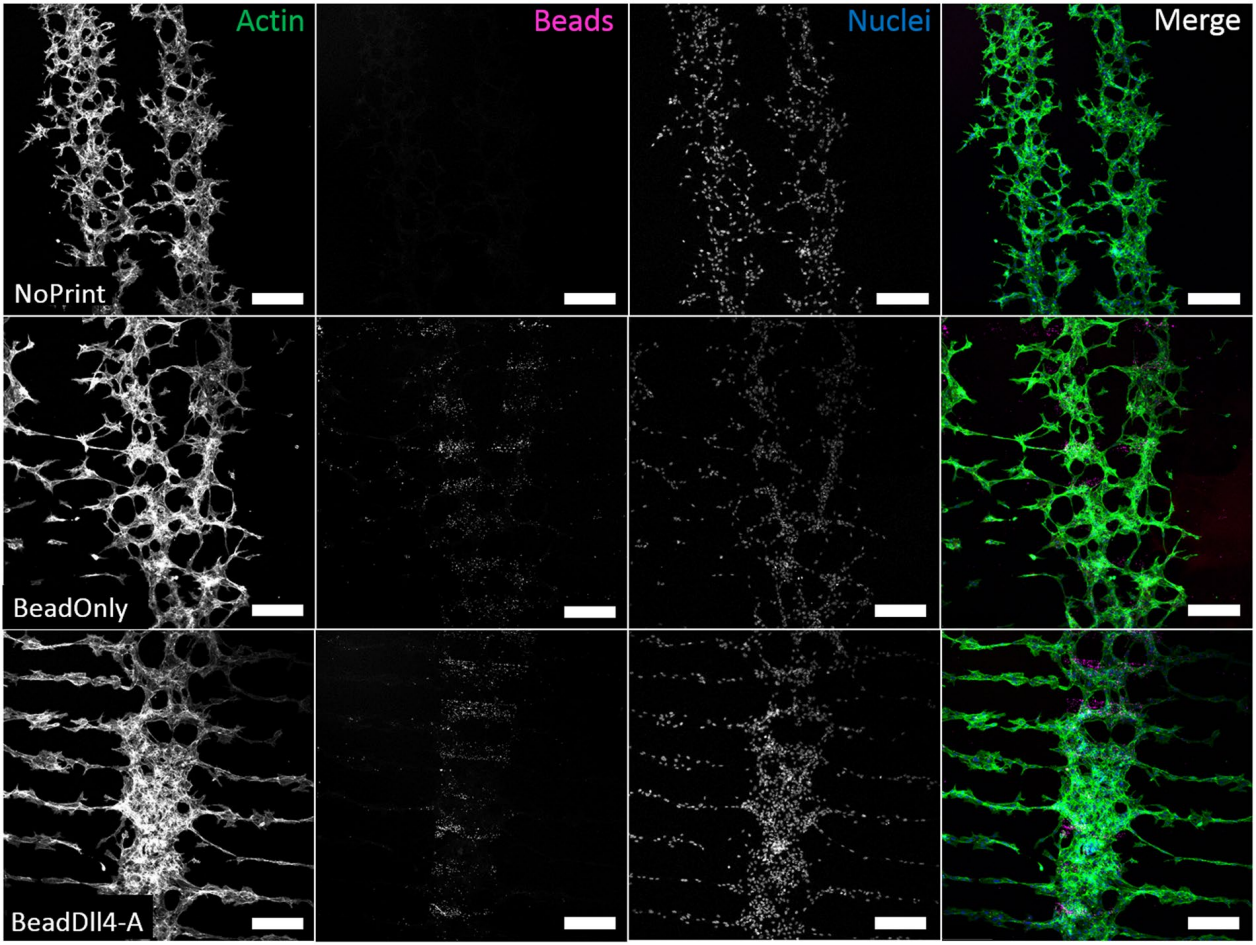
Microscopic immunofluorescent images of labelled sprouting HUVECs, in Matrigel enriched media, on top of gelatin coated glass (NoPrint, top row). Sprouting HUVECS in Matrigel enriched media on top of a line pattern of fluorescent IgG beads coated with gelatin (BeadOnly, middle row) or on a Dll4 ligand immobilized with fluorescent IgG beads line pattern coated with gelatin (BeadDll4, bottom row). First three columns contain intensity values separately. The last column contains the merged image with in green; actin stained with phalloidin, in pink; fluorescent IgG beads, and in blue; nuclei stained with DAPI. Scale bar represents 250 µm.

Figure 4 shows the comparison of the immunofluorescent microscopy images for the control conditions with the patterned bead-Dll4 ink condition. In the absence of patterned bead-Dll4 or fluorescent beads, the endothelial sprouts were shorter and had no clear direction or preference in the location of the origin of the sprouts. In the presence of patterned fluorescent beads with no Dll4 attached, the sprouts were slightly longer and less furcate, but still showed no preference in direction or location of the origin of the sprouts. When exposed to a pattern of bead-Dll4 ligands, the endothelial cells responded to the bead-Dll4 ink and formed significantly longer sprouts, directly parallel to the patterned immobilized Dll4 and demonstrated a clear preference in location of the origin of the sprouts, resulting in a pattern of endothelial sprouts in between the Dll4 ligands. These sprouts anastomosed with sprouts originating from the endothelial cells of neighbouring channels (Supplementary data Fig. 1), demonstrating the possibility of creating a spatially controlled vessel bed using patterned Dll4 ligands.

### Endothelial sprouting is spatially restricted by the bead-Dll4 pattern

**Figure 5:**
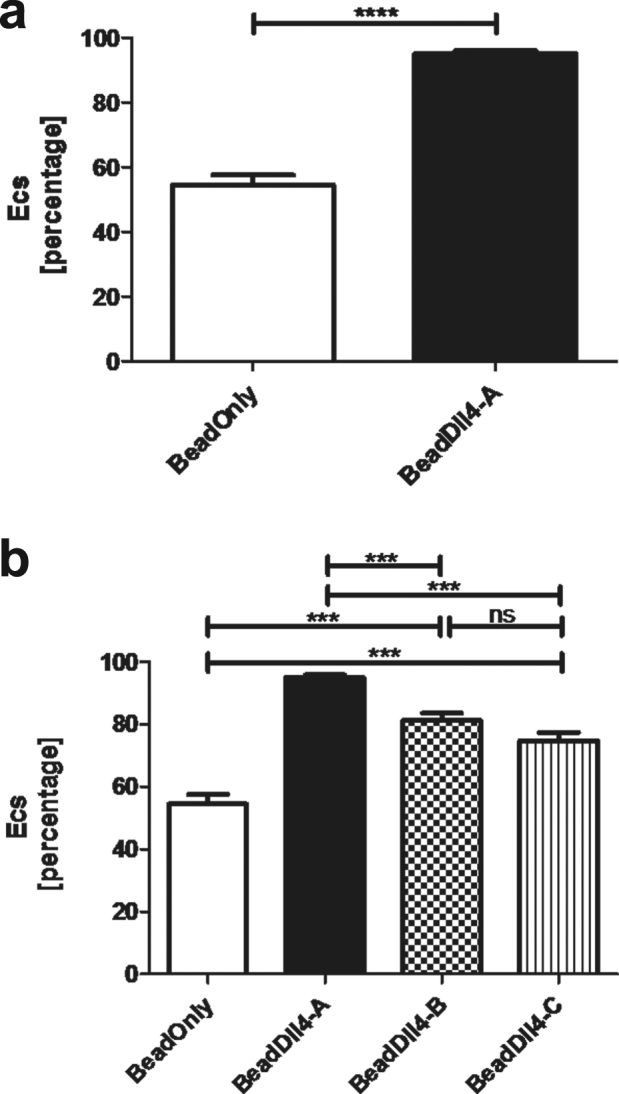
Efficiency of controlled sprouting analysed with (**a**) 89 ROIs of bead-only condition and 98 ROIs of bead-Dll4 condition. (**b**) The reproducibility of the bead-Dll4 chips resulting from the Ecs calculated with 60 ROIs of Bead-Dll4 sample B and 71 ROIs of bead-Dll4 sample (c). *p < 0.05, **p < 0.01, ***p < 0.001, ****p < 0.0001.

To quantify the level of control over the location of growing endothelial sprouts, the efficiency of controlled sprouting (Ecs) was calculated for every ROI. Equation (1) was used to determine the Ecs in percentage, with C_off_ being the number of cells not on the line of patterned bead-Dll4 (off) and C_on_ the number of cells on the line of patterned bead-Dll4 (on). Using this formula, the Ecs is equal to 100% if all the sprouting cells are located in between the patterned bead-Dll4 (off). With an Ecs of 0% negative patterning is observed, meaning the cells are located only on the patterned bead-Dll4. When the cells have no particular preference in location regarding to the bead-Dll4 pattern, the Ecs is 50%. As expected, no significant preference of the sprouts could be observed in the bead-only condition, as demonstrated by the mean Ecs of 54.5 ± 3.1% (Fig. 5a). The patterned bead-Dll4 significantly increased the Ecs to 95 ± 0.9%. These results demonstrate that the Dll4 in the printed ink pattern is the determining parameter for the spatial control over endothelial sprouting, and not the fluorescent IgG particles. It was further confirmed that the number of cells in the two areas of the ROI was similar prior to induction of sprouting and therefore any potential effect of Dll4 on proliferation did not influence the patterning of the sprouts (Supplementary data Fig. 2).

Two other chips were created and analysed in the same fashion to investigate reproducibility of the design. Again a significant difference between Ecs of the bead-Dll4 patterned chips and the bead-only control was found (Fig. 5b), with Ecs values of 81.2 ± 2.4% and 74.9 ± 2.6% observed for bead-Dll4 chips B and C respectively, leading to an average Ecs of 84.7 ± 1.86%. The data confirms that the control exerted by the pattern of bead-Dll4 over the spatial localization of the endothelial sprouting is robust and significant. The data clearly demonstrated the ability of the Dll4 ligand to locally inhibit angiogenic sprouting on the printed bead-Dll4 lines and Dll4 patterning can therefore be used to control the location of sprouting.

### Dll4 patterning increases unidirectional sprouting

**Figure 6:**
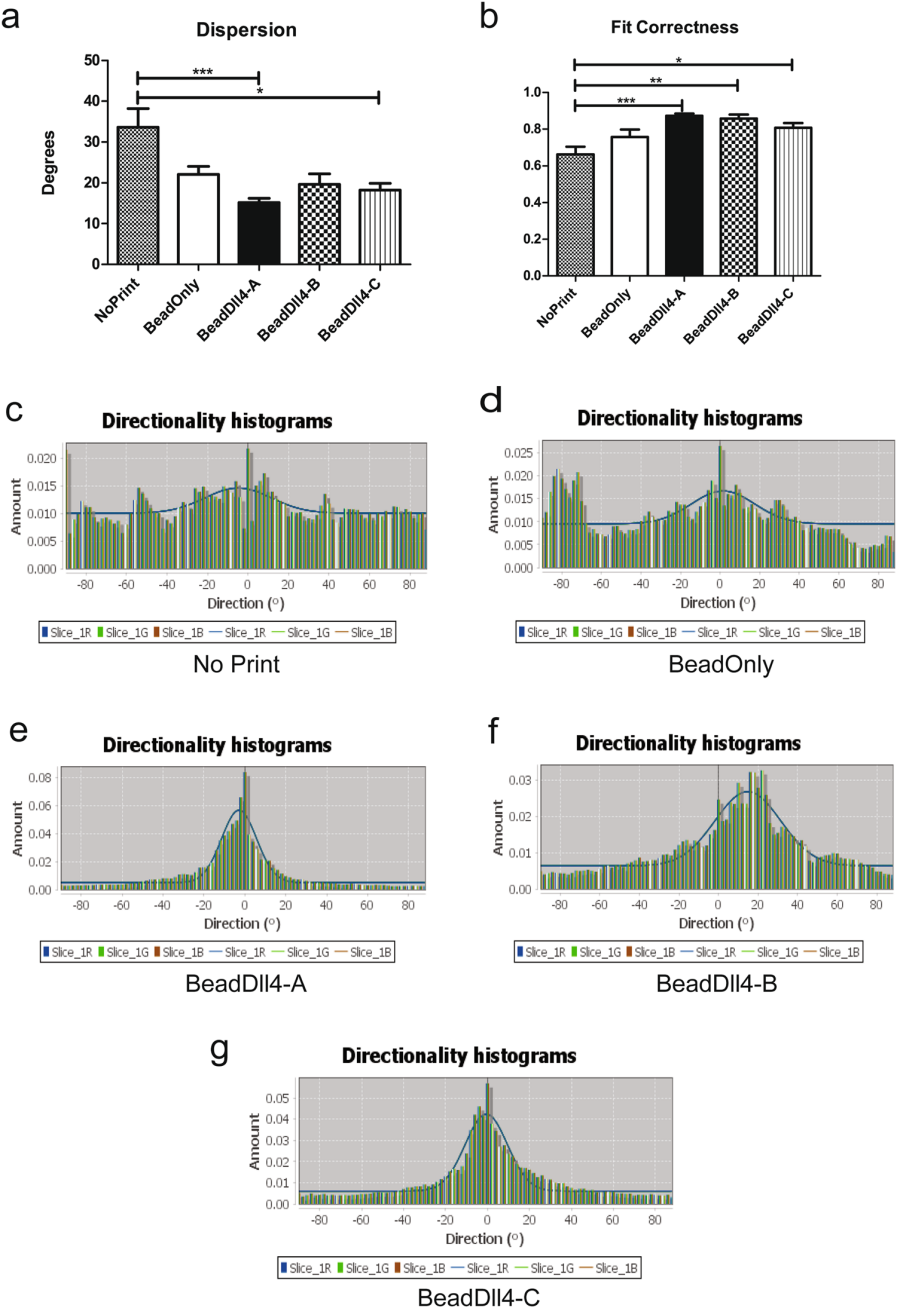
Patterned Dll4 lines result in unidirectional endothelial sprouting. Direction of 5 × 5 pixel matrices was calculated for all ROI of either side of the vessel mimicking monolayer in one microscopy image, using the Image J plugin ‘Directionality’. (**a**) The dispersion of the fitted graph through the histograms in degrees. (**b**) The correctness of this fit through the direction histograms. *p < 0.05, **p < 0.01 ***p < 0.001, ****p < 0.0001. NP n = 15, BO n = 21, BD-A n = 23, BD-B n = 13, BD-C = 22. (**c**) Histogram of the directions found in the no print condition (**d**) for the bead-only control condition and (**e**) for the patterned Dll4 condition, with similar chips BeadDll4-B and C (**f** and **g**).

The direction of the endothelial sprouts emerging from the vessel mimicking monolayer was investigated using the image J ‘Directionality’ plugin software (Fig. 6c–g). The fit dispersion of the software model was used as a parameter for the unidirectionality of the endothelial sprouting from either sides of the monolayer per immunofluorescent image. The dispersion was significantly reduced using patterned Dll4 compared to the controls, demonstrating that the control conditions had a more randomly orientated sprouting (Fig. 6a). Moreover, the correctness of the fitted direction model is increased for the patterned Dll4 condition (Fig. 6b), corroborating the significance of the dispersion data. The general direction of the sprouting however, did not differ between the controls and the patterned Dll4 condition (Supplementary data Fig. 4). As the direction perpendicular to the vessel mimicking monolayer was defined as zero degrees, the average direction of the sprouts in the patterned Dll4 condition was expected to be zero, with a small deviation. Controls were expected to have a larger dispersion. Surprisingly, the average direction was not zero for all the chips, though the average direction remained between an −5 degree and +8 degree angle (Supplementary data Fig. 4). This can be explained by the manual placement of the micro fluidic channels on top of the pattern, which might have led to a slight deviation of the supposed 90 degree angle between the channels and the pattern, resulting in different sprouting directions. It must be noted that one image analysed by the plugin contained multiple ROIs, resulting in a lower n than the number of ROIs.

Immobilization of Notch ligands to surfaces as a means to activate Notch has been done before, yet focused mostly on the influence of the Jagged ligand^14,16–19^. Dll4 has been immobilized on beads^26^ as well as been incorporated in 3D hydrogels^27^, however, here we describe the first attempt to immobilize the ligands in a spatial fashion to achieve control over vascular tissue patterning, which may be caused by our interference in the Dll4- mediated lateral inhibition process. The data in this study suggests that there is a clear difference between conditions in sprouting direction and we observe a change from random spouting to unidirectional sprouting, supporting the hypothesis that Dll4 patterning leads to more spatially controlled sprouting. This unidirectional sprouting is a direct result of the restrictions set by the patterned Dll4 beads. Recent data on Notch dose sensitivity in cell fate determination^28^ and the finding that cell-cell contact area influences the Notch pathway’s signalling strength^29,30^ support our idea that the controlled dose and location of the Dll4 ligand is responsible of forcing the endothelial phenotype. Additionally, lateral inhibition has previously been pointed out to be a possible tool for the control over cell to cell patterning^31^.

To build on the findings of our study’s, it might be suggested that engineering principles can be used to spatially control angiogenesis and vascular patterning in 2D and possibly 3D by changing the design of the Dll4 pattern. Further investigation using different geometric patterns will shed more light on the limits of the patterned Dll4 induced control. In this context, the effects of spatially patterned Jagged1 should be investigated as well, as Jagged1 and Dll4 have opposite effects on sprouting^7^. Additionally, VEGF is an interesting target for controlled angiogenesis. For example, the investigations of hydrogels containing both VEGF and Notch inhibitors in hind limb ischemia, demonstrated the important interplay between the two pathways and their contribution to neovascularization^32^. However, *in vivo* targeting of angiogenesis by these pro-angiogenic proteins and drugs does not exert control over spatial placement- and guidance of neovessels. This induces random angiogenesis in contrast with our study’s results. Research that has moved away from random vascular networks has previously relied on microfabrication techniques to create patterned (micro)vessels^33–37^. However, these approaches rely on material-, or structure induced patterning or direct cell patterning and not on biological cues^34^. Furthermore, templated micro vessels are limited by the use of 3D molds^34,37^. Our approach is unique in utilizing a patterned bio activated surface to achieve spatial control over vascularization. This implies convenient use- and application of Notch ligand patterned surfaces for direct presentation to (native) (diseased) vascular tissues to stimulate controlled sprouting in the future.

## Conclusion

In this paper we describe the development of a unique surface based method to control angiogenic sprouting. We demonstrate that µCP of Notch ligand Dll4 in patterns enables control of the location and direction of endothelial sprouting. By presenting the cells to a pre-existing alternating line pattern of micro contact printed Dll4 we obtained spatial control of angiogenesis. We show that the patterned bead-Dll4 lines are able to locally inhibit angiogenic sprouting and direct the formation- and location of new sprouts. This straight forward and surface based method of endothelial cell fate manipulation provides a stepping stone for future developments of spatial presentation of ligands on (bio)material surfaces for more controlled (neo)vascularization for tissue engineering and/or regenerative medicine applications.

## Electronic supplementary material

Supplementary Information
