# Supplementary material for: Spatial patterning of the Notch ligand Dll4 controls endothelial sprouting in vitro
Source: Sci Rep. 2018 Apr 23;8:6392. doi: 10.1038/s41598-018-24646-y (PMC5913301; doi:10.1038/s41598-018-24646-y)
Supplement: Supplementary file 1 — Supplementary Information [file 41598_2018_24646_MOESM1_ESM.pdf]

Spatial patterning of the Notch ligand Dll4 controls endothelial sprouting in *vitro*

L. A. Tiemeijer,[ab*] J-P. M. S. Frimat,[c] O. M. J. A. Stassen,[a] C. V. C. Bouten,[a] and C. M. Sahlgren[abd]

a) Department of Biomedical Engineering, Soft Tissue Engineering and Mechanobiology, ICMS Institute for Complex Molecular Systems, Eindhoven University of Technology, Eindhoven, Netherlands.

b) Faculty of Cellbiology, Åbo Akademi University, Turku, Finland

c) Department of Mechanical Engineering, Microsystems Group, ICMS Institute for Complex Molecular Systems, Eindhoven University of Technology, Eindhoven, Netherlands

d) Turku Centre for Biotechnology, University of Turku and Åbo Akademi University, Turku, Finland

Correspondence: L.A. Tiemeijer, Department of Biomedical Engineering, Eindhoven University of Technology, P.O. Box 513, 5600 MB Eindhoven, The Netherlands, +31(0)40-2475415, Email: l.a.tiemeijer@tue.nl

Supplementary information

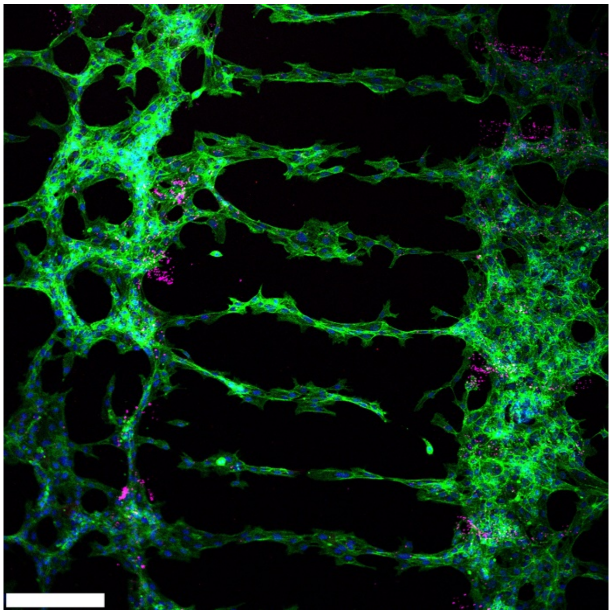

Supplementary figure 1 - Microscopic Immunofluorescent image of stained sprouting HUVECs in Matrigel enriched media on top of a Dll4 ligand immobilized with fluorescent IgG beads line pattern coated with gelatin. Endothelial sprouts originating from two (vessel mimicking) monolayers find one another in between the bead-Dll4 lines and anastomose. In green, actin stained with phalloidin. In pink, fluorescent IgG beads. In blue, nuclei stained using DAPI. Scale bar represents 250µm.

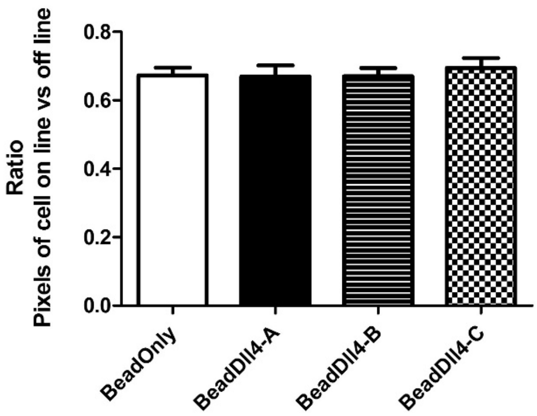

Supplementary figure 2 – To ascertain that Dll4 induced proliferation within the (vessel mimicking) monolayers prior to Matrigel induced sprouting,has no effect on the sprouting control, the amount of cells in both the area's (in between and on the bead-Dll4 lines) were counted. No difference between controls and chips were found.

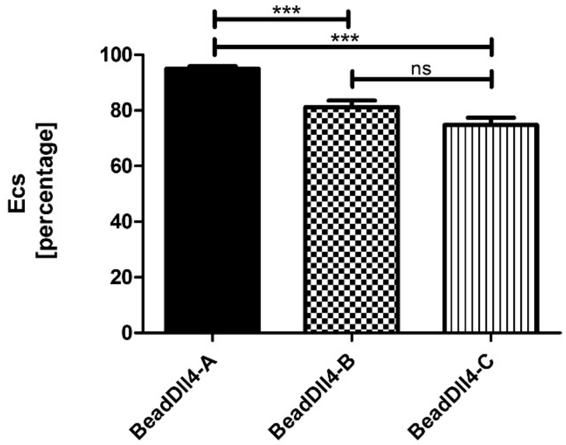

Supplementary figure 3 - Efficiency of controlled sprouting comparison between the different bead-Dll4 chips. Ecs = 81.2±2.4% and 74.9±2.6% for bead-Dll4 B and C respectively differ significantly from the Ecs = 95±0.9% of Bead-Dll4 A, but there is no difference between B and C. Bead-Dll4 A can be interpreted as the most successful chip, though B and C still show significantly more controlled sprouting than the bead-only control (Fig 5B). Data based on 98 ROIs of Bead-Dll4 sample A, 60 ROIs of Bead-Dll4 sample B and 71 ROIs of bead-Dll4 sample C. *** = $p < 0.001$.

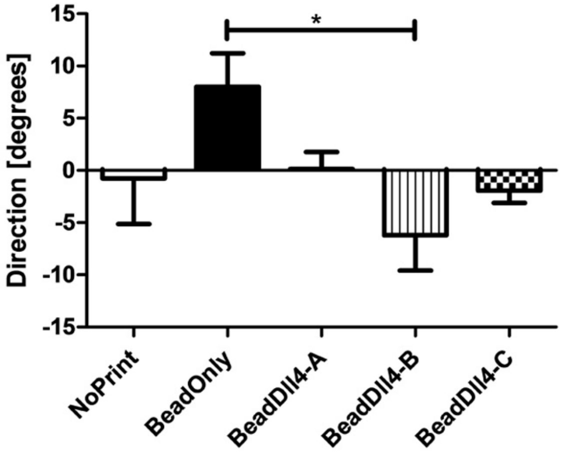

Supplementary figure 4 – Directions of the endothelial sprouts calculated for all experimental conditions using Directionality plugin of Image J. Expected direction of all the samples is around 0 degrees (horizontal). Difference in standard error of the mean is expected be decreased for bead-Dll4 conditions. * = p< 0.05, NP n=15, BO n=21, BD n=23.
